# Semaphorin 7A Aggravates Pulmonary Inflammation during Lung Injury

**DOI:** 10.1371/journal.pone.0146930

**Published:** 2016-01-11

**Authors:** Judith Marlene Roth, David Köhler, Mariella Schneider, Tiago Folgosa Granja, Peter Rosenberger

**Affiliations:** Department of Anesthesiology and Intensive Care Medicine, Tübingen University Hospital, Tübingen, Germany; Section of Pulmonary and Critical Care Medicine, UNITED STATES

## Abstract

The extent of pulmonary inflammation during lung injury ultimately determines patient outcome. Pulmonary inflammation is initiated by the migration of neutrophils into the alveolar space. Recent work has demonstrated that the guidance protein semaphorin 7A (SEMA7A) influences the migration of neutrophils into hypoxic tissue sites, yet, its role during lung injury is not well understood. Here, we report that the expression of SEMA7A is induced in vitro through pro-inflammatory cytokines. SEMA7A itself induces the production of pro-inflammatory cytokines in endothelial and epithelial cells, enhancing pulmonary inflammation. The induction of SEMA7A facilitates the transendothelial migration of neutrophils. In vivo, animals with deletion of SEMA7A expression showed reduced signs of pulmonary inflammatory changes following lipopolysaccharide challenge. We define here the role of SEMA7A in the development of lung injury and identify a potential pathway to interfere with these detrimental changes. Future anti-inflammatory strategies for the treatment of lung injury might be based on this finding.

## Introduction

Acute lung injury (ALI) develops in response to pneumonia, major surgery or prolonged mechanical ventilation and is associated with a high mortality rate [1]. A critical step during the early stages of lung injury is the migration of neutrophils from the vascular compartment into the alveolar space. As a result of this process, a self-propagating inflammation develops within the alveolar space. The severity of the associated symptoms is determined by the extent of alveolar inflammation and is of key importance for the outcome of affected patients [2]. The infiltration of neutrophils and the development of inflammation within the alveolar space are controlled by classical paradigms through the chemokine system [3, 4]. However, recent work has also demonstrated a significant role for neuronal guidance protein signaling in the control of neutrophil migration and the orchestration of acute inflammation [5–7].

We have shown recently that a member of the class of neuronal guidance proteins and a member of the semaphorin family proteins, semaphorin 7A (SEMA7A), induces the migration of neutrophils into hypoxic tissue sites [8]. The semaphorins are a large family of secreted and cell surface proteins that modulate neurite extension. SEMA7A also induces the production of cytokines in macrophages and monocytes, which plays a significant role during the effector phase of the inflammatory immune response [9, 10]. Furthermore, SEMA7A also stimulates cytoskeletal reorganization in melanocytes and monocytes, which translates into cell morphology changes that can result in the spreading and migration of these cell types [11–13]. Previous work has indicated that SEMA7A might also initiate inflammation during seawater aspiration induced lung injury [14]. The etiology of lung injury is frequently either an inflammatory process within the lung or an inflammatory process that hits the lung as a secondary organ, leading to lung injury as a sequela of the primary process.

We aimed to characterize the role of SEMA7A during lung injury and its role in pulmonary inflammation. We report here that SEMA7A significantly influences the extent of pulmonary inflammation.

## Materials and Methods

### RT-qPCR

Human microvascular endothelial (HMEC-1) and lung epithelial (A549) cells were grown to confluence. Cells were subjected to 100ng/ml TNF-α or 20ng/ml IL-6 for indicated time periods. Additionally cells were stimulated with increasing concentration of TNF-α or IL-6 for 4h. For RNA extraction we used the peqGOLD TriFast^™^ (Peqlab; Germany; Erlangen) standard procedure due to manufacture instruction. For cDNA synthesis we used the iScript kit from Bio-Rad (Bio-Rad; Germany; Munich). Semiquantitative analysis for human SEMA7A was performed using real-time PCR. The primer set consisted of the sense primer 5’-CTC AGC ATC CAG CGA CAT-3’ and the antisense primer 5’-ACA GGG GCA CTA TCC ACA AG-3’. Human β-actin (sense primer, 5'-AGA GGC GTA CAG GGA TAG CA 3' and antisense primer, 5'-GGA GAA AAT CTG GCA CCA CA-3') and human 18S (sense primer, 5'-GTA ACC CGT TGA ACC CCA TT-3' and antisense primer, 5'-CCA TCC AAT CGG TAG TAG CG-3') were used as controls. Transcriptional analysis of murine SEMA7A was performed using the sense primer 5'-GTG GGT ATG GGC TGC TTT TT-3' and the antisense primer 5'-CGT GTA TTC GCT TGG TGA CAT-3'. Samples were controlled using murine 18S with the following set of primers: sense 5'-GTA ACC CGT TGA ACC CCA TT-3' and antisense primer 5’-CCA TCC AAT CGG TAG TAG CG-3’. In a subset of experiments, human cell cultures were exposed to 100ng/ml recombinant SEMA7A (R&D Systems, Cat. No. 2068-S7-050) for indicated time periods. Transcriptional analysis for TNF-α and IL-6 expression was performed using the following primer sets: TNF-α; sense primer 5’-GAG CTG CCC CTC AGC TTG-3’ and the antisense primer 5’-ATC TTC TCG AAC CCC GAG TGA-3’ and IL-6; sense primer 5’-CAC CAG GCA AGT CTC CTC AT-3’ and the antisense primer 5’-GAC AGC CAC TCA CCT CTT CA-3’. In additional experiments to analyze SEMA7A receptor expression in murine lung tissue the following primers were used: Plexin C1; sense primer 5’-TTA GGA AGG AGG CGA AGA GA-3’ and the antisense primer 5’-ACA GAG ACG CCA ATG ACA AG-3’; integrin β1; sense primer 5’-GGT GTC GTG TTT GTG AAT GC-3’ and the antisense primer 5’-TCC TGT GCA CAC GTG TCT-3’; integrin αv; sense primer 5’-CAA GCT CAC TCC CAT CAC-3’ and antisense primer 5’-GGG TGT CTT GAT TCT CAA AGG G-3’ and α1; sense primer 5’-CCT TCC CTC GGA TGT GAG TCA-3’ and the antisense primer 5’-AAG TTC TCC CCG TAT GGT AAG A-3’.

### Protein analysis

Subsequent homogenizations of HMEC-1, A549 or murine lung tissue isolated proteins were resuspended in RIPA buffer. The determination of protein quantity, was performed according to the standard protocol of the BCA Protein Assay Kit (Thermo Scientific, Cat. No. 23225). The samples were normalized for protein levels and loaded in SDS polyacrylamide gels. After blotting on PVDF membranes following antibodies were used in human samples: monoclonal mouse anti-IL-6 antibody (abcam, Cat. No. ab66231), polyclonal rabbit Anti-TNF-alpha (abcam, Cat. No. ab66579), polyclonal rabbit Anti-SEMA7A antibody (abcam, Cat. No. ab23578) and for the control of loading conditions a monoclonal mouse β-Aktin antibody (Santa Cruz Biotechnology; Cat. No. sc-81178). In Western blots for murine samples, we used a polyclonal rabbit Anti-SEMA7A antibody (abcam, Cat. No. ab23578) and for loading control a polyclonal rabbit GAPDH antibody (Santa Cruz Biotechnology; Cat. No. sc-25778). A goat anti-rabbit IgG antibody (Santa Cruz, Cat. No. sc-2007) or a goat anti-mouse IgG antibody (Santa Cruz, Cat. No. sc-2008) respectively conjugated with alkaline phosphatase (AP) were used for detection. Bands were detected through color reaction of the AP-conjugated antibodies with a NBT/BCIP solution. Blots were developed in this solution until bands were visible. Densitometry was performed using Image J 1.44p software.

### Semaphorin ELISA

SEMA7A ELISAs were performed according to the manufacturer’s instructions (ELISA Kit Uscn, Life Science Inc.; Kit for SEMA7A human; Cat. No. SEB448Hu). In cell experiments, 100 μl of supernatant was used after stimulation with TNF-α (100 ng/ml) for 4 h. Supernatants of vehicle-stimulated cells served as controls.

### Isolation of human neutrophils and transendothelial migration assay (TEM)

After approval by the Institutional Review Board of Tübingen University Hospital (ethics committee) and after written informed consent was obtained from each person studied blood was withdrawn and polymorphonuclear leukocytes (PMN) isolated from whole blood obtained. Isolated PMNs were employed in transendothelial migration assays.

For studying transmigration, HMEC-1 cells were grown confluently on the apical site of permeable Transwell inserts (pore size 3μm). Isolated neutrophils from peripheral blood of healthy donors were used to test transmigration over HMEC-1 monolayer. PMNs were preincubated for 90 minutes at 37°C with 1μg/ml human recombinant SEMA7A (R&D Systems), appropriate recombinant human IgG_1_ Fc (R&D Systems) or PBS^-^ only (Sigma Aldrich, Cat. No. 8537). IgG_1_ Fc treatment and PBS^-^ only served as control experiments. As chemoattractant 10 ng/ml fMLP (N-formyl-methionine-leucine-phenylalanine) in HBSS+ was added to the lower compartment of the transwells. The migration assay and the quantification of PMNs based on MPO measurement were performed as described previously [7].

### Murine model of pulmonary inflammation

Approval of the Institutional review Board and the Regierungspräsidium Tübingen was obtained. *SEMA7A*^*-/-*^ (The Jackson Laboratory, Maine, USA) and littermate controls were matched according to sex, age and weight and exposed to LPS inhalation experiments for 45 min as previously described [15]. After 4 hours animals were anesthetized and the bronchoalveolar lavage (BAL) was received through tracheal incision and instillation of 3 x 0.6ml 0.9% saline solution. Finally lungs were harvested for further examination.

### Cellcounts

Cellcounts were performed using 10μl BAL diluted in 10 ml CASY ton solution (Roche, Cat. No. 05 651 808 001) via CASY Model TT cell counter (Roche Diagnostics GmbH, Mannheim, Germany).

### Measurement of BAL cytokine concentration

All cytokines determined in BAL were measured according to standard protocols by ELISA (TNF-α, IL-6, IL-1β, KC and MIP-2 from R&D Systems; CXCR2 from Uscn, Life Science Inc.).

### Semaphorin 7A Reporter Assay

Promoter analysis and identification of potential NF-κB-binding sites were performed using the search engines MatInspector and BioBase. Vector PGL4.17-expressing sequences corresponding to the full-length SEMA7A promoter—either native (SEMA7A_FL_, -1041 to +29, 1.069 bp) or containing mutations in one of the following potential NF-κB binding sites: SEMA7A-NF-κB_4_ (bp -1000 to -1008), SEMA7A-NF-κB_3_ (bp -818 to -826), SEMA7A-NF-κB_2_ (bp -675 to -683) and SEMA7A-NF-κB_1_ (bp -188 to -196)–were purchased from GeneArt (Regensburg, Germany). New sequences were checked using the above-described search engines to ensure that no new transcription factor-binding sites were generated. Transfection of cells was accomplished using the standard conditions of the PolyFect Transfection Reagent (QIAGEN, Cat. No. 301105). Luciferase activity was assessed using a Steady-Glo Luciferase Assay System (Promega, Madison, USA) after cells were stimulated with TNF-α (100ng/ml; 24h). All firefly luciferase activity was normalized to the total protein content.

### NF-κB Chromatin Immunoprecipitation Assay (ChIP)

ChIP assay was performed according to the manufacturer’s instructions (Chromatin Immunoprecipitation Kit CHP1, Sigma Aldrich, USA). The ChIP-qualified antibody of interest was the NF-κB antibody NF-κB p105/p50 (abcam Cat. No. ab-7971-1; input = 1 μg). To induce NF-κB binding A549 cells were treated with TNF-α (100ng/ml; 4h). The following PCR primers for SEMA7A DNA detection were used: NF-κB binding site at position -188 (= SEMA7A-NF-κB_1_): 5’-AGT GGA ACT GAG GCC CAG AGA-3’, 5’-TCT CGC CTC ACT GGC TTT CC-3’; at position -675 (= SEMA7A-NF-κB_2_): 5’-TTG CGG GCA GAG ATC CCC-3’, 5’-TCC CAG CCA GCA GCC TT-3’; at position -818 (= SEMA7A-NF-κB_3_): 5’-GCA CGT AGT TCT GAG AGG AGG-3’, 5’-CAG CTT AAG GGG ATC TCT GCC-3’; at position -1000 (= SEMA7A-NF-κB_4_): 5’-TCA CCT CTA CTC CTT CCT CGG-3’, 5’-TCT CTC TAG ACC AGA CAG GGC-3’.

### Immunofluorescence staining

HMEC-1 and A549 cells were grown on chamber slides (Thermo Scientific, Cat. No. 177399), stimulated for 4h with 100ng/ml TNF-α, 20ng/ml IL-6 or 100ng/ml rhSEMA7A and fixed for 10 minutes in acetone and methanol 1:1. After being permeabilized with 1% TritonX cells were washed with PBS and blocked with 5% BSA in PBS for 1h. Cells were stained with goat polyclonal anti-SEMA7A (R&D, Cat. No. AF2068), rabbit polyclonal Anti-IL-6 (abcam, Cat. No. ab6672) rabbit polyclonal Anti-TNF-alpha antibody (abcam, Cat. No. ab66579). As secondary antibody following antibodies were used: polyclonal donkey anti-goat IgG Alexa Fluor 488 (Thermo Scientific, Cat. No. A-11055), and polyclonal goat anti-rabbit IgG Alexa Flour 488 (Thermo Scientific, Cat. No. A-11008). For structural presentation monoclonal mouse β-Actin (ACTBD11B7) (Santa Cruz Biotechnology; Cat. No. sc-81178) antibody was used. Visualization of β-Aktin was done using goat anti-mouse IgG Alexa Flour 594 (Thermo Scientific, Cat. No. A-11005) as secondary antibody. Nuclei were counterstained with Roti-Mount FluorCare DAPI (Carl Roth GmbH, Cat. No. HP20.1). For murine histological stainings, lungs were embedded in paraffin. Immunofluorescence staining of murine lungs were performed as described previously [8] using a polyclonal rabbit Semaphorin 7a antibody (abcam, Cat. No. ab31449). Goat anti-rabbit IgG Alexa Flour 488 (Thermo Scientific, Cat. No. A11008) was used as secondary antibodies. Imaging was performed with an Axiophot Zeiss microscope (Zeiss, Germany) using a digital camera with AxioVision 4.8 software.

### Immunohistological staining

Immunohistochemistry of neutrophils in lung sections was performed with the Vectastain ABC Kit (Linaris, Mannheim, Germany). After blocking with avidin blocking solution (Vector Labs, Burlingame, USA) for 1 h at room temperature, sections were incubated with a monoclonal rat Ly-6B.2 Alloantigen Antibody 7/4 (AbD Serotec, Cat. No. MCA771GA) or rat IgG control (Santa Cruz, Cat. No. sc-2026) at a dilution of 1:1000 overnight at 4°C. Tissue sections were then incubated with biotinylated rabbit anti-rat IgG (Vector Labs, Cat. No. BA-4000) for 30 min followed by Vectastain ABC reagent (Vector Labs, Cat. No. PK-4000) for 30 min and then developed using Histogreen (Liniaris, Cat. No. E109) as substrate. Counterstaining was performed using nuclear fast red (Linaris, Cat. No. H-3403). Images were then processed with a Leitz DM IRB microscope (Leica). All images were analyzed with the software AxioVision v4.8.2

### Quantification of pulmonary myeloperoxidase activity (MPO)

Pulmonary infiltration by PMNs was measured as previously described [16]. Briefly, quantification of MPO was performed by using 50μl of BAL. BAL samples were incubated light protected for 25 minutes at 37°C with 50μl citrate-puffer and 100μl ABTS. Quantification of MPO was determined in the infiniteM200PRO TECAN Reader at 405 nm.

### Data analysis

Statistical significance was determined using one-way analysis of variance (ANOVA) followed by post hoc test. Student’s *t*-test was used for 2 groups comparison. A value of P < 0.05 was considered statistically significant.

## Results

### Semaphorin 7A is induced during lung injury

**Fig 1:**
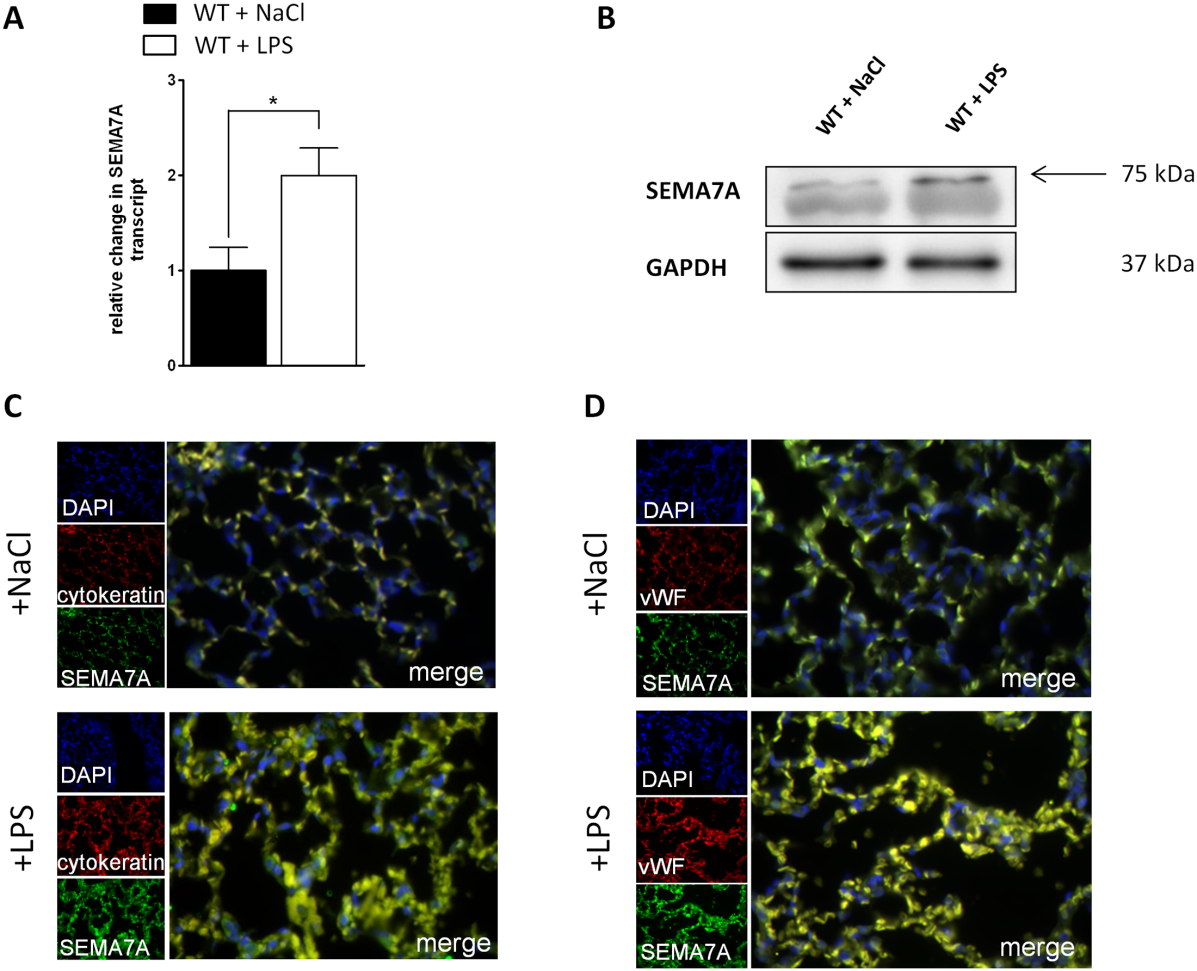
SEMA7A is induced during lung injury. Lungs from WT animals were harvested 4 hours after exposure to either NaCl or LPS inhalation. **A)** Relative mRNA of SEMA7A in lung tissue determined by qPCR following LPS inhalation (n≥5/group). **B)** Western blot analysis of SEMA7A (n = 3/group) and **C)** Immunofluorescence analysis of SEMA7A (green), cytokeratin as epithelial cell marker (red) in the lung following either NaCl or LPS inhalation, cell DNA marker DAPI (blue) and merge (yellow) (n = 4/group). **D)** Immunofluorescence analysis of SEMA7A (green), von Willebrand Factor (vWF) as endothelial cell marker (red) in the lung following either NaCl or LPS inhalation, cell DNA marker DAPI (blue) and merge (yellow) (n = 4/group). For negative controls see S1 Fig (all the data are expressed as the mean±SEM, *P < 0.05, as indicated).

We first evaluated whether SEMA7A expression was changed during acute pulmonary inflammation in vivo. We exposed WT animals to LPS inhalation (500μg/ml). We observed a significant induction of SEMA7A mRNA and protein expression in pulmonary tissue (Fig 1A and 1B). This could also be observed when pulmonary tissue was stained for the presence of SEMA7A by immunofluorescence (Fig 1C, S1 Fig). SEMA7A target receptor expression was evaluated within the lung using RT-PCR (S2 Fig).

### SEMA7A is induced in vitro through pro-inflammatory cytokines

**Fig 2:**
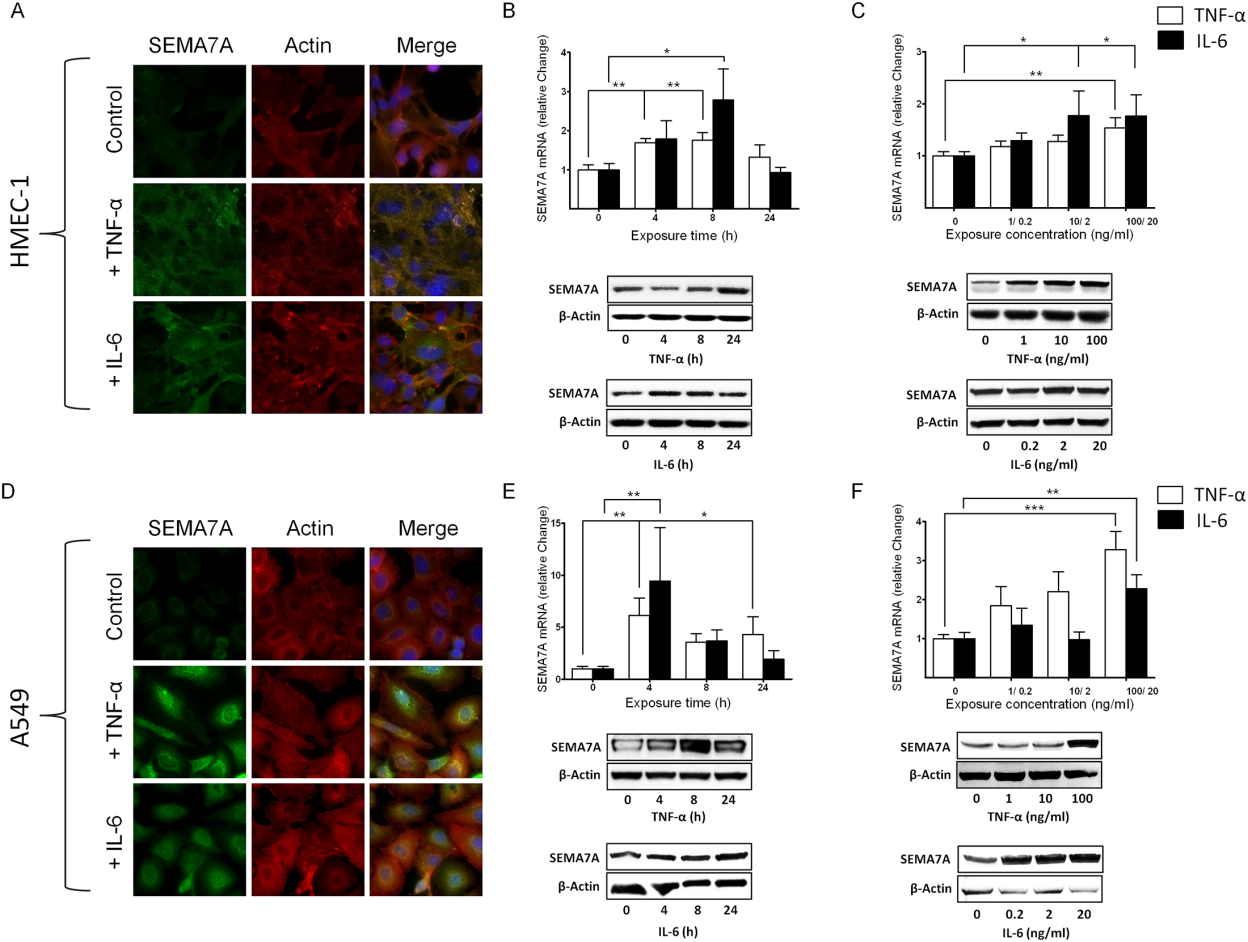
Regulation of SEMA7A during inflammation. Endothelial HMEC-1 and alveolar epithelial A549 cells were exposed to pro-inflammatory cytokines **A)** SEMA7A immunofluorescence in endothelial HMEC-1 following stimulation with TNF-α (100ng/ml) and IL-6 (20ng/ml) for 4 hours with SEMA7A (green), Actin (red), cell DNA marker DAPI (blue) and merge (yellow). **B)** Relative change in SEMA7A-mRNA and protein expression after exposure of HMEC-1 cells to TNF-α and IL-6 for the indicated time periods. **C)** Relative change in SEMA7A-mRNA and protein expression after exposure of HMEC-1 cells to increasing concentrations of TNF-α and IL-6. **D)** SEMA7A immunofluorescence in epithelial A549 cells following stimulation with TNF-α and IL-6 for 4 hours with SEMA7A (green), Actin (red), cell DNA marker DAPI (blue) and merge (yellow). **E)** Relative change in SEMA7A-mRNA and protein expression after exposure of epithelial A549 cells to TNF-α and IL-6 for the indicated time periods. **F)** Relative change in SEMA7A-mRNA and protein expression after exposure of epithelial A549 cells to increasing concentrations of TNF-α and IL-6 (all the data are expressed as the mean±SEM, *P < 0.05; **P < 0.01; ***P < 0.001 as indicated, n≥4/ group, one representative picture/ blot of three is demonstrated).

Next, we tested whether the induction of SEMA7A could also be observed in vitro. We exposed endothelial HMEC-1 cells grown to confluence to pro-inflammatory cytokines TNF-α and IL-6. Following immunofluorescent labeling of SEMA7A, we found an induced fluorescent signal for SEMA7A in response to TNF-α and IL-6 (Fig 2A, S3 Fig). The induction of SEMA7A was confirmed through transcriptional and protein analysis (Fig 2B). When exposing HMEC-1 to increasing concentrations of TNF-α or IL-6, we observed a dose-dependent induction of SEMA7A using Western blot analysis (Fig 2C, S4 Fig). In pulmonary alveolar epithelial cells, baseline expression of SEMA7A was very low without stimulation. Following exposure to TNF-α and IL-6, we found a significantly stronger signal for SEMA7A compared to non-stimulated controls (Fig 2D). Again, we performed dose- and time-dependent stimulation with TNF-α or IL-6 in the A549 cells. We found a significant induction of SEMA7A over time (Fig 2E, S4 Fig) and in response to increased concentration of TNF-α or IL-6 (Fig 2F, S4 Fig).

### SEMA7A induces the expression of pro-inflammatory cytokines

**Fig 3:**
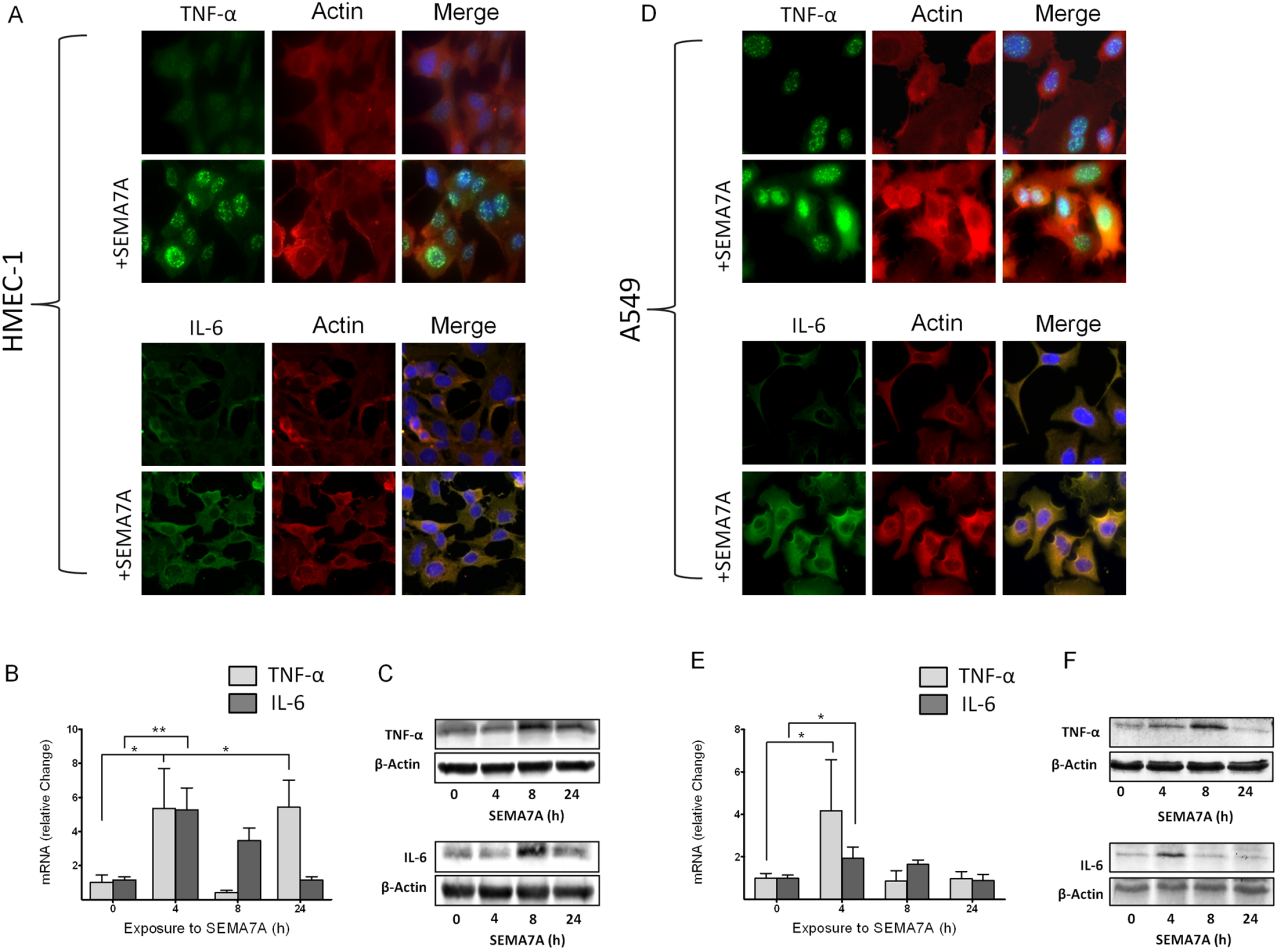
SEMA7A induces the expression of pro-inflammatory cytokines. Endothelial HMEC-1 and alveolar epithelial A549 cells were exposed to human recombinant SEMA7A. **A)** TNF-α and IL-6 immunofluorescence in endothelial HMEC-1 following stimulation with SEMA7A for 4 hours, TNF-α or IL-6 (green), Actin (red), cell DNA marker DAPI (blue) and merge (yellow). **B)** Relative change in TNF-α and IL-6 mRNA after exposure of HMEC-1 cells to SEMA7A at different time points. **C)** Relative change in TNF-α and IL-6 protein expression after exposure of HMEC-1 cells to SEMA7A for up to 24 hours. **D)** TNF-α and IL-6 immunofluorescence in epithelial A549 cells following stimulation with SEMA7A for 4 hours, TNF-α or IL-6 (green), Actin (red), cell DNA marker DAPI (blue) and merge (yellow). **E)** Relative change in TNF-α and IL-6 mRNA after exposure of epithelial A549 cells to SEMA7A at different time points. **F)** Relative change in TNF-α and IL-6 protein expression after exposure of A549 cells to SEMA7A for up to 24 hours (all the data are expressed as the mean±SEM, *P < 0.05; **P < 0.01; as indicated, n≥4/ group, one representative picture/ blot of three is demonstrated).

To further evaluate the function of SEMA7A in the context of inflammation, we exposed HMEC-1 and A549 cell lines to human recombinant SEMA7A and measured the expression of TNF-α and IL-6. To exclude an unspecific effect of SEMA7A we performed control experiments but did not find an unspecific effect of the IgG_1_ Fc portion of recombinant SEMA7A on cytokine production (S5 Fig). Immunohistochemical staining of HMEC-1 cells following exposure to SEMA7A revealed an induction of immunofluorescence for TNF-α and IL-6 (Fig 3A, S3 Fig). Evaluation of mRNA using RT-PCR and Western blot analysis confirmed this upregulation (Fig 3B and 3C). We also performed these experiments in A549 cells and demonstrated an induction of SEMA7A immunofluorescence following TNF-α and IL-6 exposure (Fig 3D). We also observed an induction of pro-inflammatory cytokines TNF-α and IL-6 mRNA and protein expression following exposure to SEMA7A (Fig 3E and 3F, S6 Fig).

### SEMA7A promoter is influenced by NF-κB

**Fig 4:**
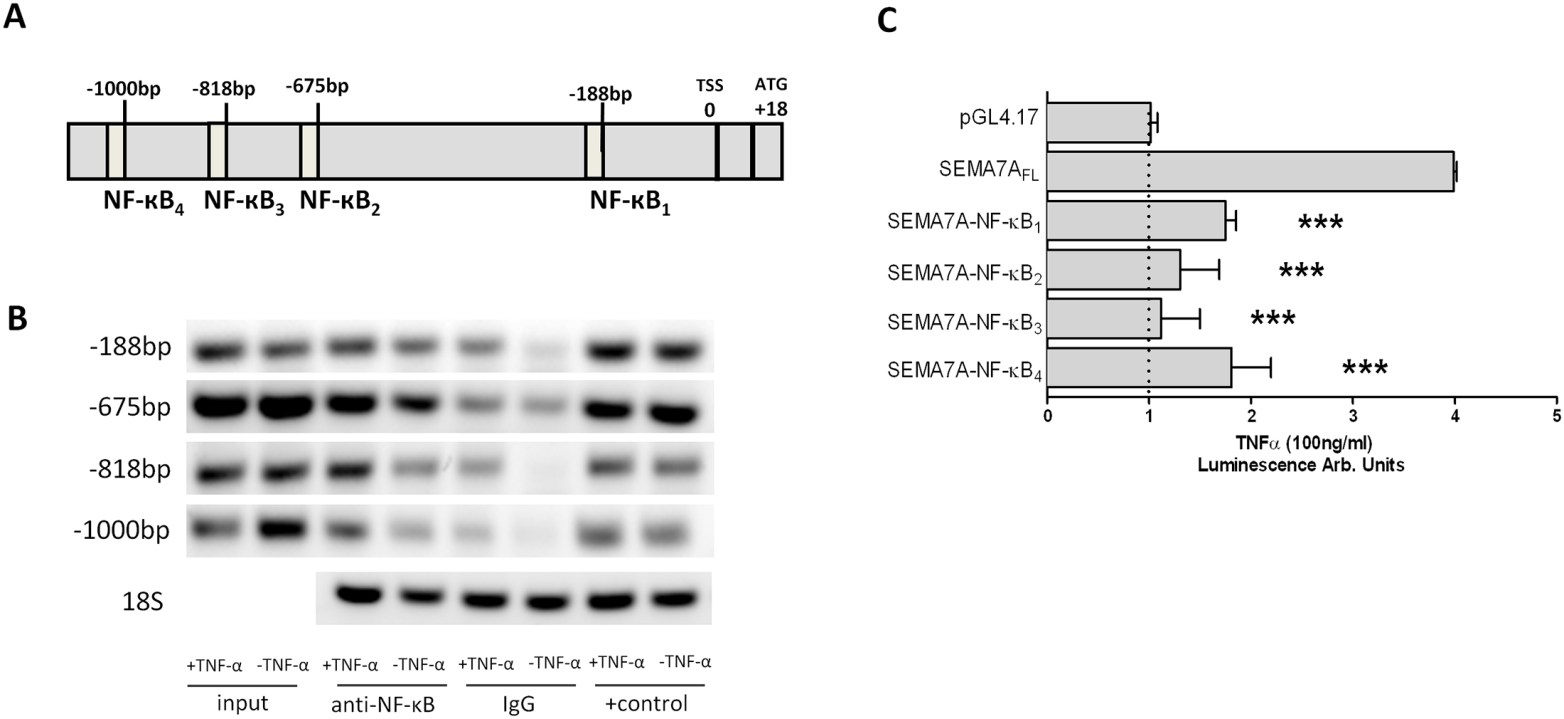
Role of NF-κB in the regulation of SEMA7A. **A)** Graphic representation of the putative SEMA7A promoter. Four potential NF-κB binding elements were identified in the upstream sequence relative to the transcription start site (TSS). **B)** ChIP assay was employed to examine NF-κB binding to the human SEMA7A promoter in A549 monolayers after exposure to TNF-α. **C)** A549 cells were transfected with the SEMA7A reporter plasmid and site-directed mutation of each NF-κB binding site was performed. Empty reporter vector (pGL4.17) was used as a negative control and SEMA7A full length (SEMA7A-NF-κB_FL_) represents the SEMA7A promoter construct without site-directed mutation. All data are expressed as mean±SEM compared to SEMA7A-NF-κB_FL_, ***P < 0.001 as indicated, n = 3/ group, one representative blot of three is demonstrated).

In order to gain specific insights into the mechanism of SEMA7A regulation during inflammation, we used available public databases [17] to analyze the full-length cDNA. The transcription start site of human SEMA7A was identified at position +18 relative to the first codon (Fig 4A). Four binding sites for NF-κB required to constitute an inflammatory response were detected in the SEMA7A promoter. They are located at approximate positions -1000 bp, -818 bp, -675 bp and -188 bp relative to the transcription start site. We used chromatin immunoprecipitation to determine whether the SEMA7A promoter binds NF-κB. Chromatin-immunoprecipitation analysis of nuclei derived from A549 cells showed a prominent band for NF-κB binding in samples following stimulation with TNF-α (Fig 4B). To investigate the functional role of these binding sites, we employed luciferase reporter constructs containing the putative full-length SEMA7A promoter (SEMA7A FL, from TSS to -1017). As shown in Fig 4C, A549 cells transiently transfected with this construct showed a 4.0-fold increase in luciferase activity after exposure to TNF-α compared to the control (pGL4.17). To investigate which of the NF-κB binding sites was responsible for this induction, we performed site-directed mutations of each individual binding site. As seen in Fig 4C, lack of any NF-κB binding site resulted in a reduction of the observed SEMA7A induction.

### SEMA7A is cleaved in endothelial and epithelial cells and induces the transmigration of neutrophils

**Fig 5:**
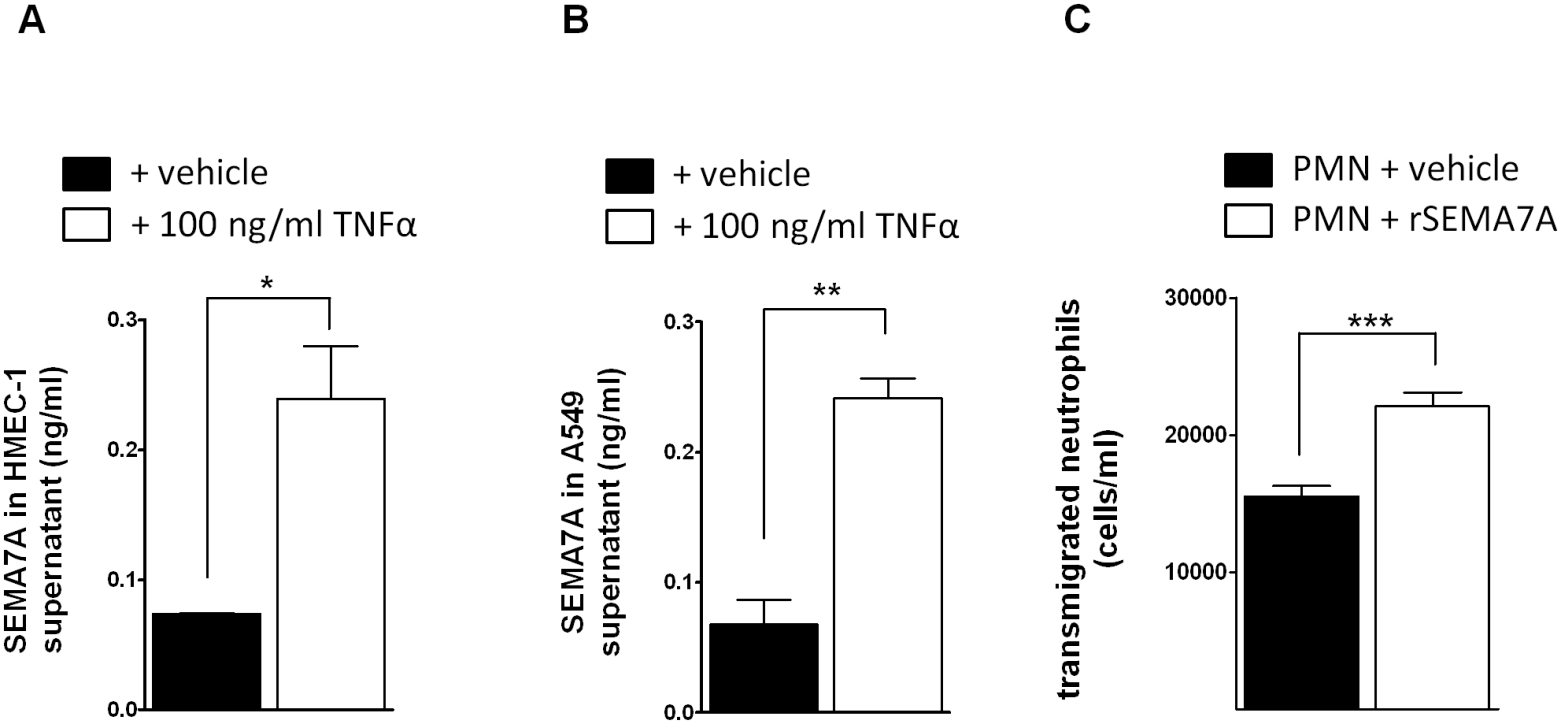
SEMA7A is released during inflammatory stimulation from endothelial and epithelial cells to induce PMN transmigration. **A)** Endothelial HMEC-1 monolayers were exposed to inflammatory stimulation with TNF-α and SEMA7A was measured in the supernatant (n = 3/group). **B)** Epithelial A549 monolayers were exposed to inflammatory stimulation with TNF-α and SEMA7A was measured in the supernatant (n = 3/group). **C)** Human recombinant SEMA7A increased transendothelial migration of neutrophils (n = 5/group). PMNs were exposed to SEMA7A and the migration of neutrophils was measured after 90 min (all the data are expressed as the mean±SEM, **P < 0.01; ***P < 0.001 as indicated).

Next, we tested whether SEMA7A is released from endothelial cells and epithelial cells following exposure to inflammatory cytokines. We exposed HMEC-1 and A549 cells to TNF-α for 4 hours and observed an increase of SEMA7A in the supernatant (Fig 5A and 5B). We have shown previously that overexpression of SEMA7A in endothelial cells results in the transendothelial migration of neutrophils [8]. Here, we exposed neutrophils to free human recombinant SEMA7A. We found that neutrophils demonstrated increased transendothelial migration following exposure to free SEMA7A (Fig 5C, see control S7 Fig).

### SEMA7A-deficient mice demonstrate dampened lung injury after LPS exposure

**Fig 6:**
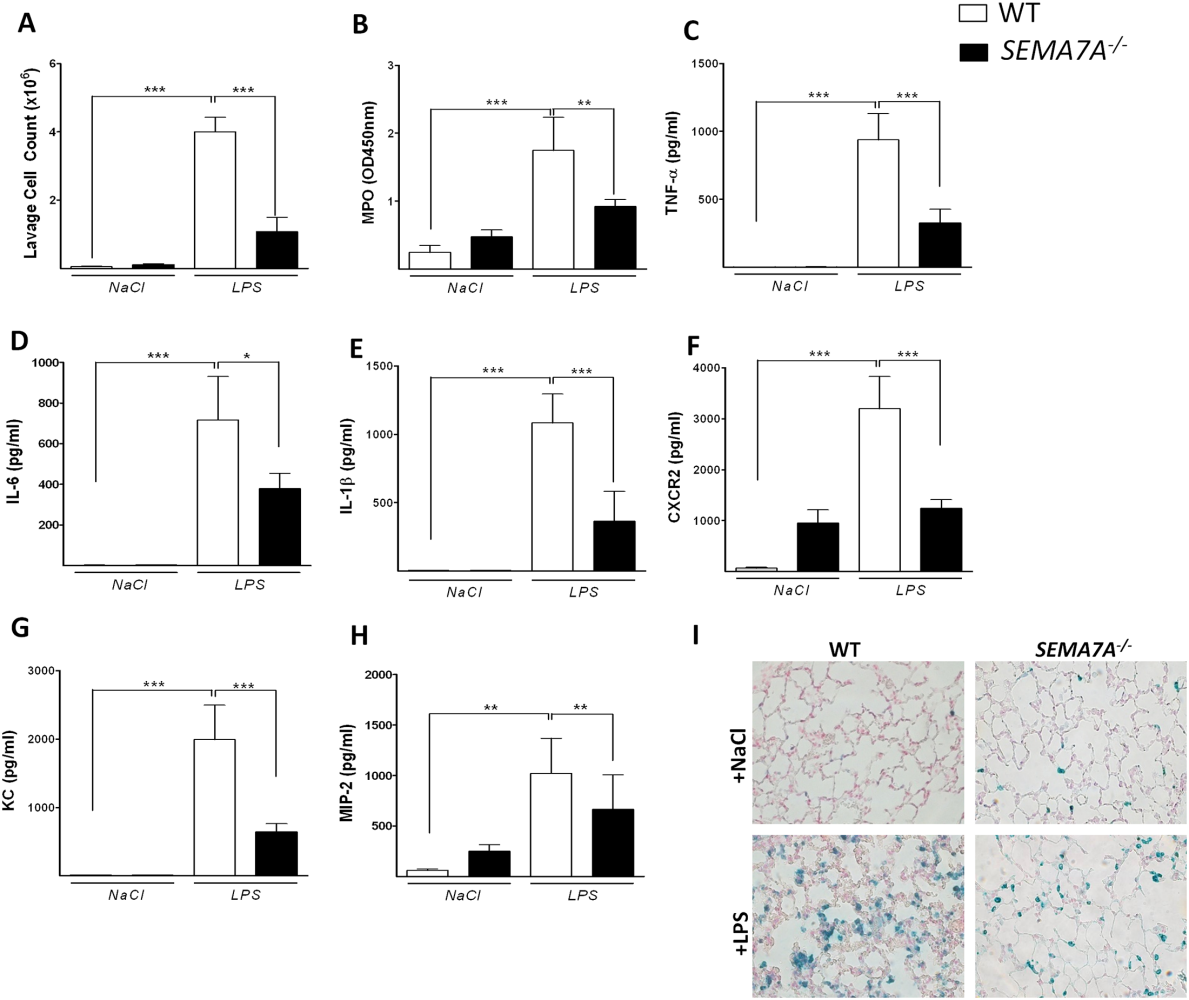
*SEMA7A*^*-/-*^ mice show attenuated pulmonary inflammation during lung injury. WT and *SEMA7A*^*-/-*^ animals were exposed to either NaCl or LPS inhalation and the degree of inflammation was determined 4 hours later **A)** Cell counts and **B)** MPO activity in the BAL of WT and *SEMA7A*^*-/-*^ mice. **C)** TNF-α **D)** IL-6 **E)** IL-1β **F)** CXCR2 **G)** KC and **H)** MIP-2 (pg/ml) concentrations measured in the BAL of WT and *SEMA7A*^*-/-*^ animals **I)** Staining of PMNs in histological sections of pulmonary tissue from WT and *SEMA7A*^*-/-*^ animals exposed to either NaCl or LPS. A representative picture out of three different lungs is shown (all the data are expressed as the mean±SEM, *P < 0.05; **P < 0.01; ***P < 0.001 as indicated, n≥6/ group).

We next investigated the role of SEMA7A in a LPS-induced lung injury model. Wild-type (WT) and semaphorin 7a-deficient (*SEMA7A*^*-/-*^) mice were exposed to either NaCl or LPS inhalation. After 4 hours, we performed bronchoalveolar lavage and determined the cell count and inflammatory factors in the BAL. We found a significantly reduced number of leukocytes in the BAL of *SEMA7A*^*-/-*^ mice compared to littermate controls (Fig 6A). We also found reduced activity of myeloperoxidase within the BAL (Fig 6B). The expression of the inflammatory cytokines and chemokines TNF-α, IL-6, Il-1β, CXCR2, KC and MIP-2 was also decreased within the alveolar space of *SEMA7A*^*-/-*^ mice (Fig 6C–6H). We also found a significantly reduced number of neutrophils within the pulmonary tissue of *SEMA7A*^*-/-*^ mice compared to controls using the Vectastain technique (Fig 6I, S8 Fig).

## Discussion

We have previously shown that SEMA7A influences PMN migration during hypoxia [8]. We have now extended this work to the inflammatory condition of lung injury and evaluated the role of SEMA7A during this common clinical condition. We demonstrated that SEMA7A is of significant importance for the development of lung injury. SEMA7A is not only induced during inflammation but also enhances inflammation itself, propagating the detrimental changes that are necessary for the development of clinical pathology. Thus, interference with the SEMA7A pathway holds therapeutic potential for the treatment of inflammatory changes associated with lung injury.

The induction of SEMA7A has been previously demonstrated during tissue hypoxia. The induction of SEMA7A expression in endothelial cells was confirmed in a study by Zhang et al. using a model of lung injury through seawater aspiration [14]. Previously, we have described the induction of SEMA7A by hypoxia inducible factor 1 α (HIF-1α), and Zhang et al. showed in their model that HIF-1α also mediates the induction of SEMA7A [14]. In the present study, we now show that SEMA7A is also induced by NF-κB. To confirm this experimentally, we precisely mutated each binding site for NF-κB. Previous work has demonstrated a close relationship between HIF-1α and NF-κB; therefore, SEMA7A could be induced by both transcription factors [18]. NF-κB expression is necessary for the induction of HIF-1α, and NF-κB is also responsive to tissue hypoxia via hydroxylase enzymes [19]. Therefore, it is possible that SEMA7A is induced via HIF-1α during lung injury, as extensive tissue inflammation results in inflammatory tissue hypoxia. Most likely, the induction of NF-κB is present before HIF-1α, as the inflammatory insult to the lung is most likely present, but further work will be needed to tease apart the close relationship between NF-κB and HIF-1α on the SEMA7A promoter region.

In addition, we showed that not only is SEMA7A induced during inflammation but also that SEMA7A induces a pro-inflammatory response. This demonstrates the close interplay between the induction of SEMA7A and the enhancement of inflammation. This finding is in accordance with previous investigations into the role of SEMA7A. SEMA7A can induce the production of cytokines in macrophages and monocytes, which play a significant role during the effector phase of the inflammatory immune response [9, 10]. In a study by Holmes et al., the authors demonstrated that SEMA7A is potent stimulator of cytokine production in monocytes. In addition, the authors demonstrated that SEMA7A significantly impacts the chemotaxis of monocytes [9]. A study by Suzuki et al. showed that SEMA7A is a crucial regulator of the T-cell response in the immune system. Here, SEMA7A also stimulated the production of cytokines and was described as critical for the effector phase of an inflammatory immune response [10]. Interestingly, it might be possible to short sequences of generated from SEMA7A binding to the Plexin C1 receptor as a possible anti-inflammatory strategy [20, 21]. Yet the implications of short peptide fragments generated from the SEMA7A need to be addressed in further work in the future. Our results demonstrated here are in accordance with this previous evidence on the role of SEMA7A in cytokine production and immune cell migration. Both of these functions are critically important for the development of lung injury and support the role of SEMA7A during lung injury that we describe here. This study is the first report to include the use of SEMA7A knockout mice in a model of lung injury. This deletion of SEMA7A ameliorates the detrimental effects of pulmonary inflammation. The study by Zhang et al. did not use genetic deletion of SEMA7A in their model of seawater aspiration and is therefore limited as to the conclusion of the role of SEMA7A during lung injury. We have extended this approach in our study. The genetic deletion of SEMA7A results in significant reduction of pulmonary inflammation.

In conclusion, our results highlight the importance of SEMA7A for the control of an acute inflammatory response and for the treatment of acute lung injury. The interference with the described pathway holds the potential to reduce the conditions associated with acute inflammation in the future. As such, further description of the role of SEMA7A during lung injury and a transfer into clinical evidence should be pursued in the future to understand the role of SEMA7A.

## Supporting Information

S1 Fig Appropriate negative and IgG controls for immunofluorescence staining of pulmonary tissue in WT animals. (TIF)

S2 Fig Expression SEMA7A target receptors in lung tissue evaluating the mRNA for receptors plexin C1, integrin β1, integrin αv and integrin α1. Tissue was taken from WT animals (C57BL/6) mice (n≥3).
(TIF)

S3 Fig Appropriate negative and IgG controls for immunofluorescence staining in cultured A549 **A)** and HMEC-1 **B)** cells for SEMA7A and actin. TNF-α and IL-6 cytokine stainings in A549 **C)** and HMEC-1 **D)** cells.
(TIF)

S4 Fig Densitometric quantification of protein analysis (Western Blotings) of target protein SEMA7A relative to housekeeping (β-Actin). SEMA7A protein in HMEC-1 **(A** and **B)** or A549 **(E** and **F)** cells exposed to 100ng/ml TNFα or 20 ng/mL IL-6) for 0, 4, 8 and 24 hours were quantified by densitometry (n≥3). In addition, HMEC-1 **(C** and **D)** or A549 **(G** and **H)** cells were exposed to increasing concentrations of TNFα (0, 1, 10 and 100 ng/mL) or IL-6 (0, 0.2, 2.0 and 20 ng/mL) for 4 hours and SEMA7A quantified by densitometry (n≥5).
(TIF)

S5 Fig HMEC-1 (A and B) or A549 (C and D) cells were exposed to PBS only or IgG_1_ Fc for 4 hours to compare the expression of TNFα (A and C) or IL-6 (B and D) mRNA. (TIF)

S6 Fig Densitometric quantification of protein analysis (Western Blots) of target proteins TNFα and IL-6 relative to housekeeping (β-Actin). TNFα or IL-6 protein of HMEC-1 **(A and B)** or A549 **(C and D)** cells exposed to 100ng/ml SEMA7A for 4 hours were quantified by densitometry (n≥3).
(TIF)

S7 Fig A) In control experiments PMNs were pretreated with PBS only or with IgG_1_ Fc for 30 minutes before starting a transendothelial migration assay. The migration of neutrophils was measured after 90 min (n = 14).
(TIF)

S8 Fig Appropriate negative and IgG controls for histological staining identifying the presence of PMNs in the lungs of WT and *SEMA7A*^*-/-*^ animals. (TIF)
