# Supplementary figures and images for: Semaphorin 7A Aggravates Pulmonary Inflammation during Lung Injury
Source: PLoS One. 2016 Jan 11;11(1):e0146930. doi: 10.1371/journal.pone.0146930 (PMC4720127; doi:10.1371/journal.pone.0146930)

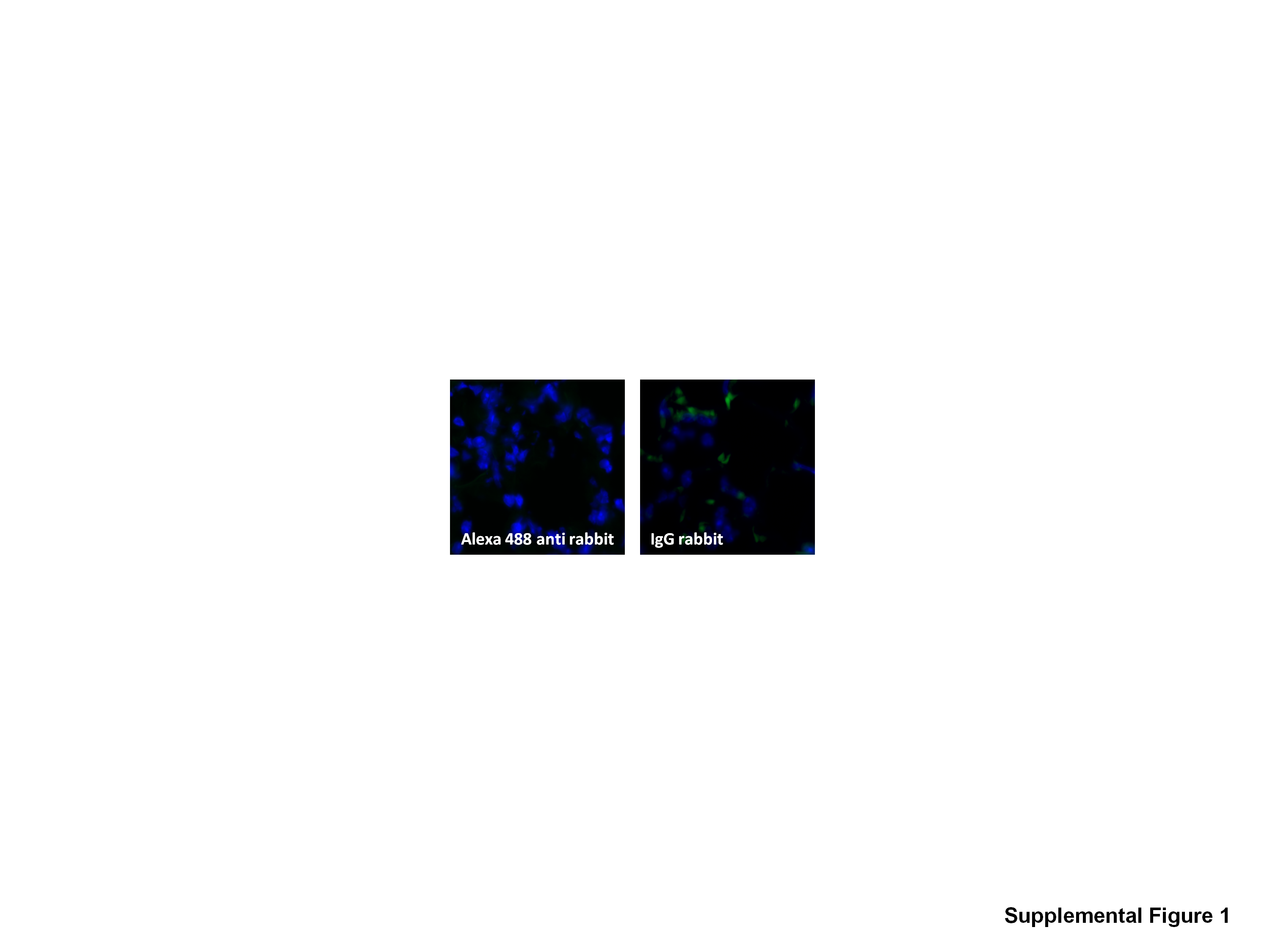

Supplement: S1 Fig — (TIF) [file pone.0146930.s001.TIF]

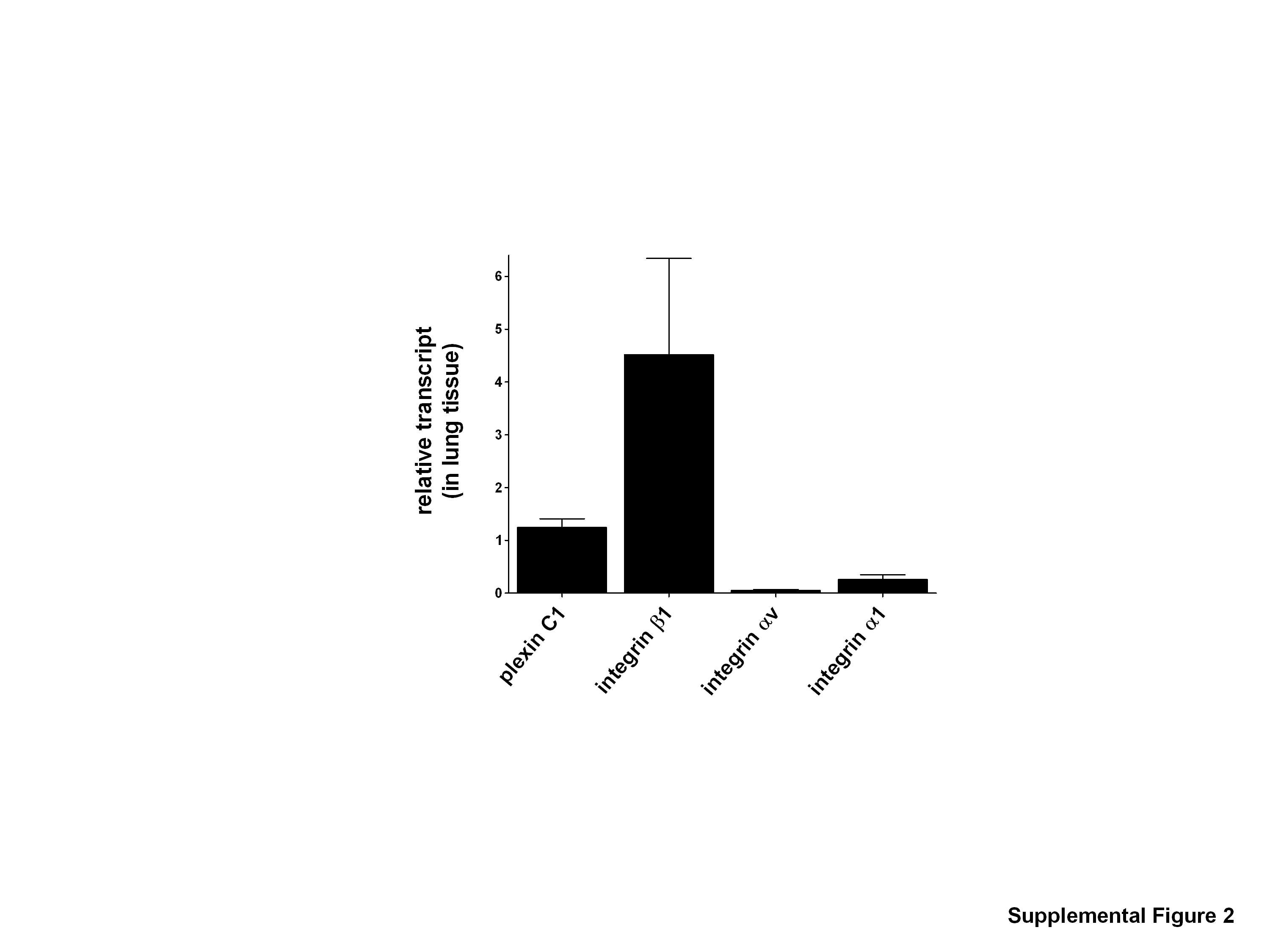

Supplement: S2 Fig — Tissue was taken from WT animals (C57BL/6) mice (n≥3). (TIF) [file pone.0146930.s002.TIF]

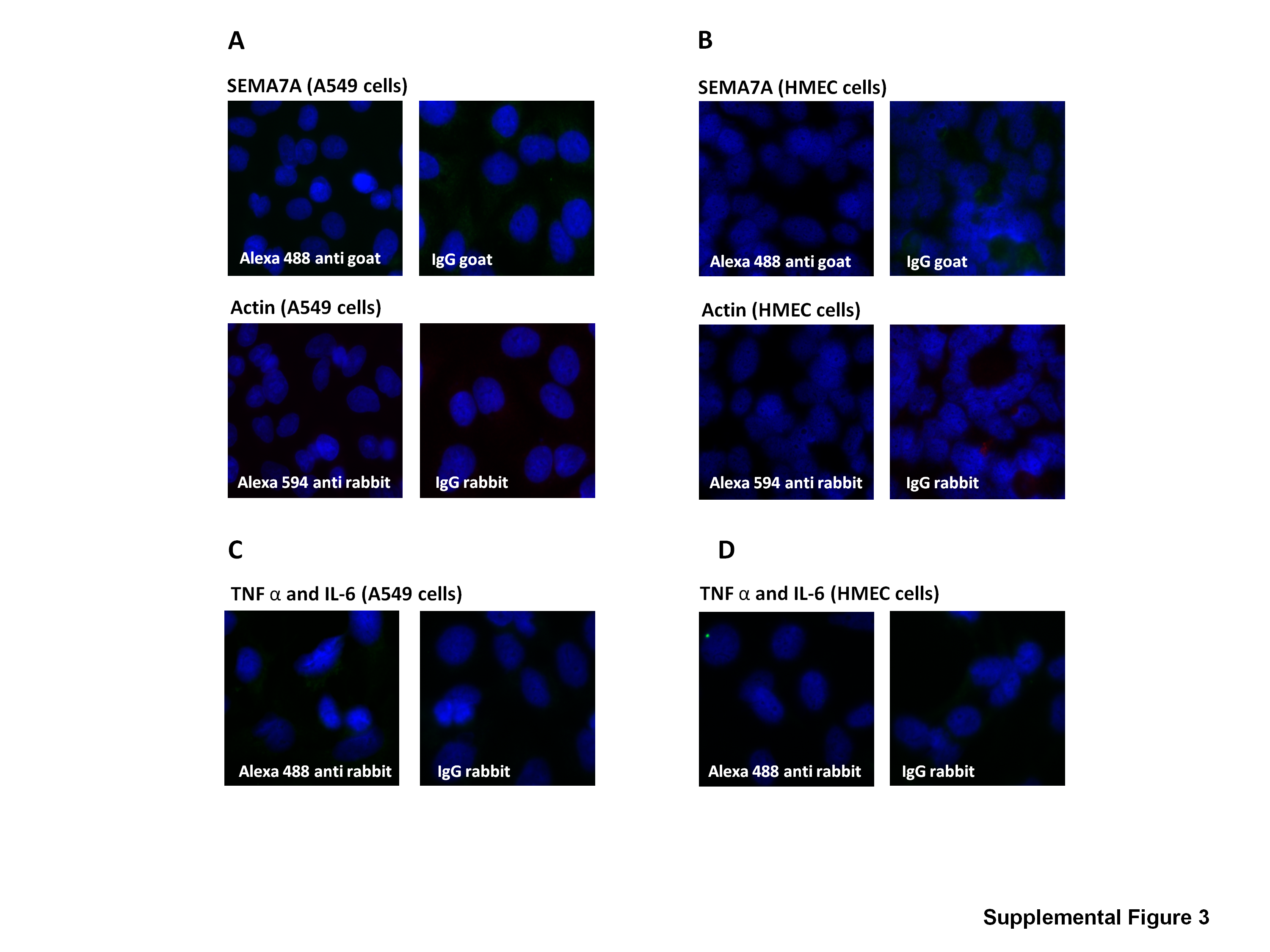

Supplement: S3 Fig — Appropriate negative and IgG controls for immunofluorescence staining in cultured A549 A) and HMEC-1 B) cells for SEMA7A and actin. TNF-α and IL-6 cytokine stainings in A549 C) and HMEC-1 D) cells. (TIF) [file pone.0146930.s003.TIF]

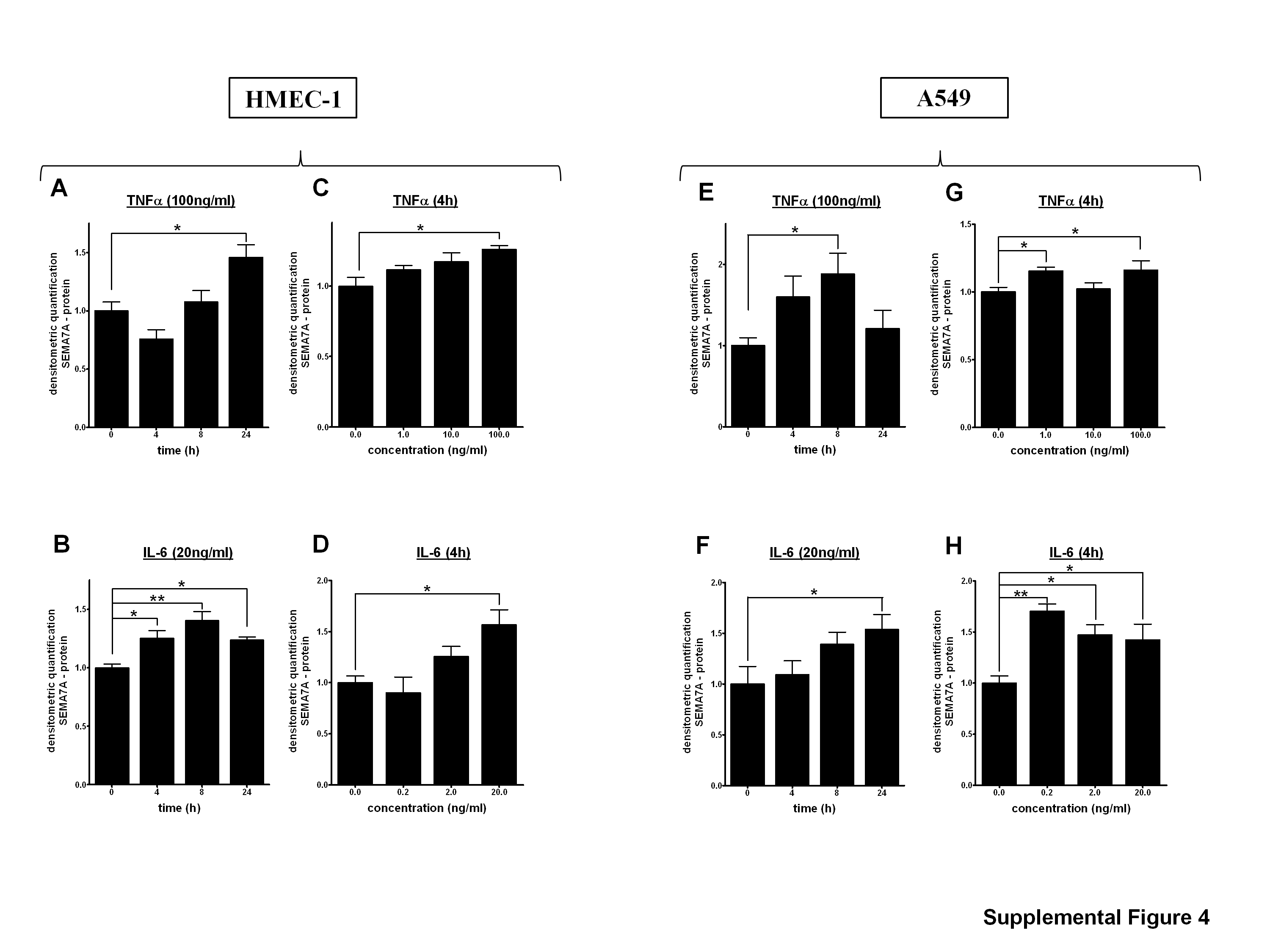

Supplement: S4 Fig — SEMA7A protein in HMEC-1 (A and B) or A549 (E and F) cells exposed to 100ng/ml TNFα or 20 ng/mL IL-6) for 0, 4, 8 and 24 hours were quantified by densitometry (n≥3). In addition, HMEC-1 (C and D) or A549 (G and H) cells were exposed to increasing concentrations of TNFα (0, 1, 10 and 100 ng/mL) or IL-6 (0, 0.2, 2.0 and 20 ng/mL) for 4 hours and SEMA7A quantified by densitometry (n≥5). (TIF) [file pone.0146930.s004.TIF]

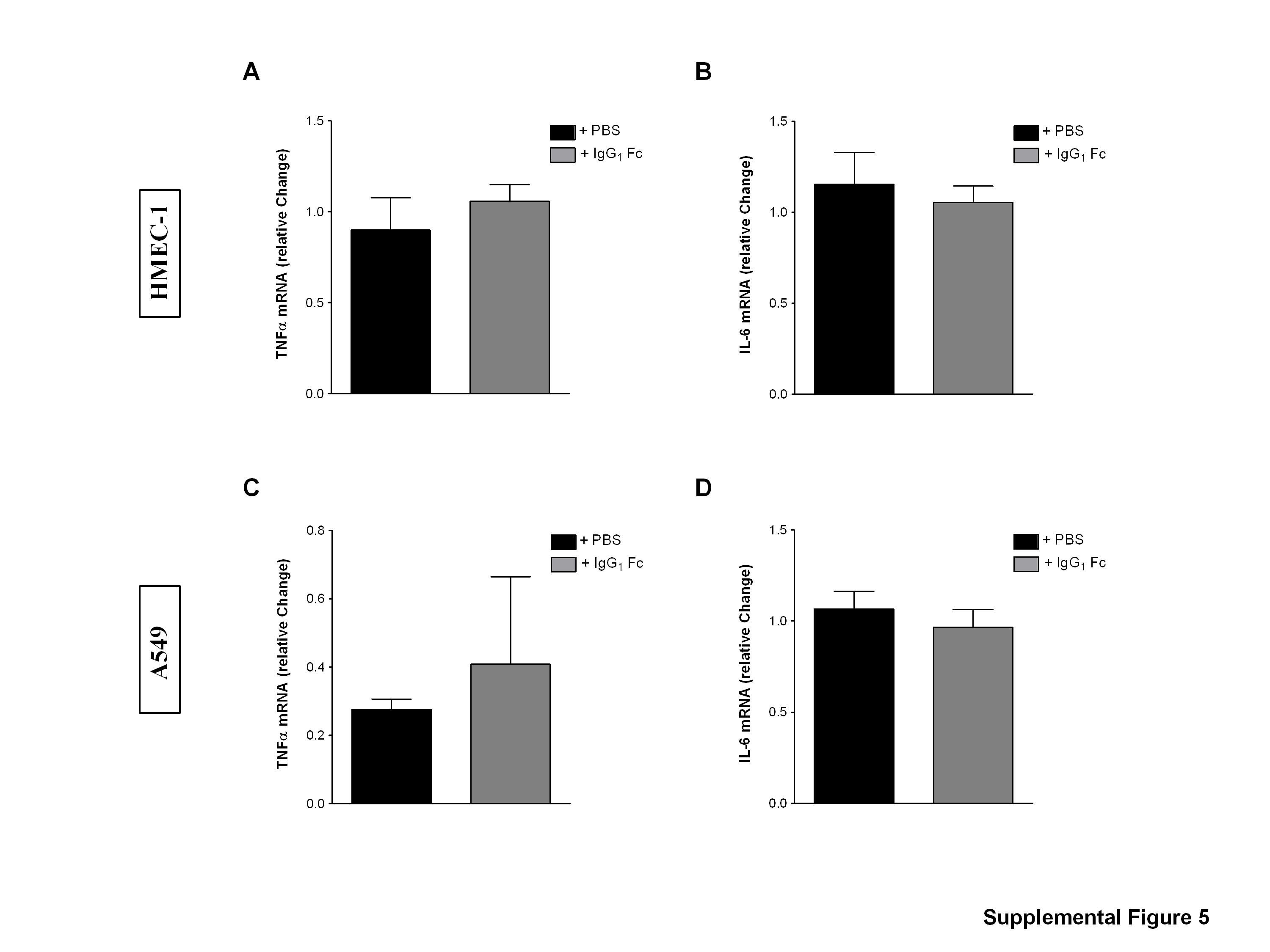

Supplement: S5 Fig — (TIF) [file pone.0146930.s005.TIF]

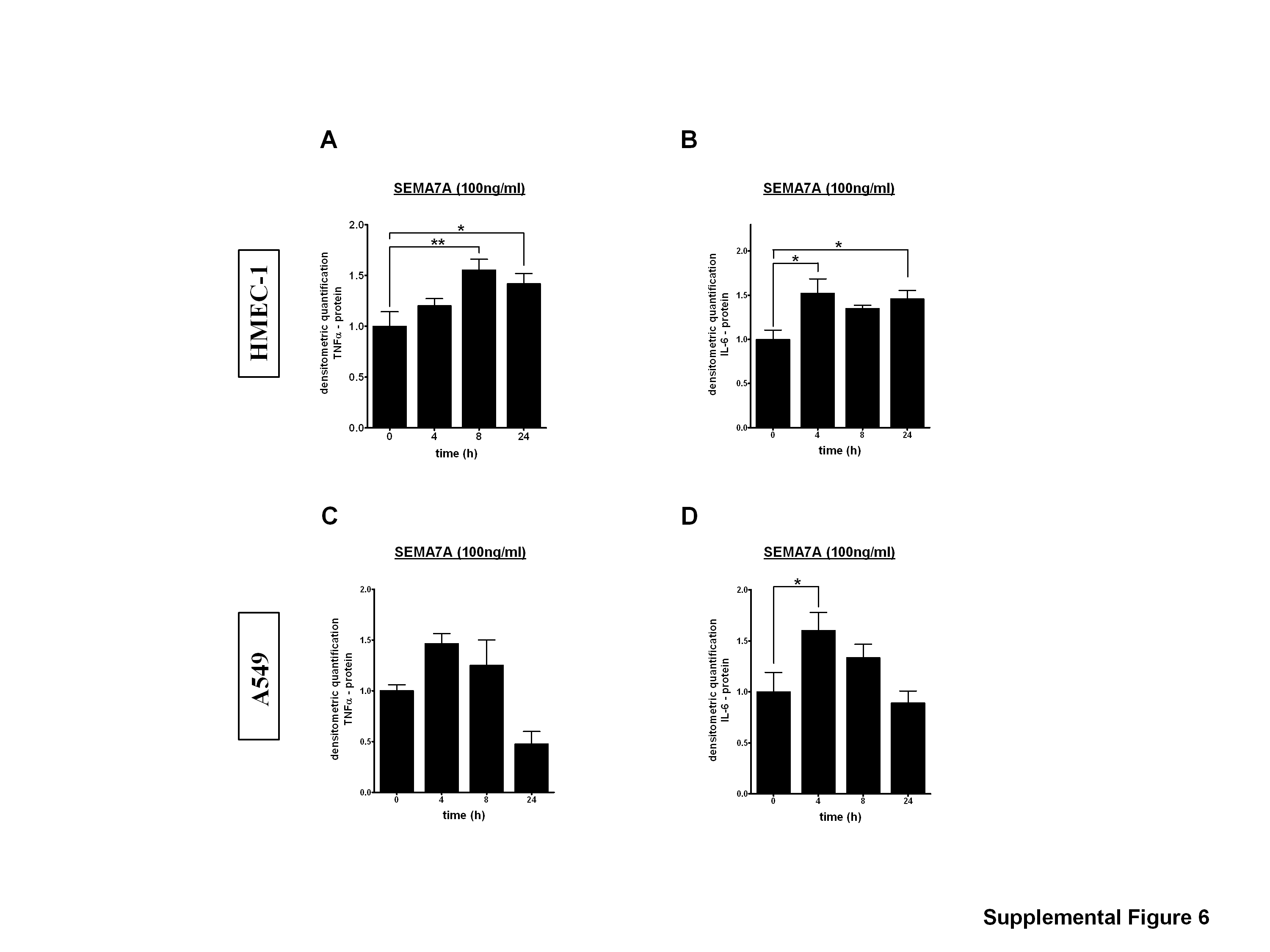

Supplement: S6 Fig — TNFα or IL-6 protein of HMEC-1 (A and B) or A549 (C and D) cells exposed to 100ng/ml SEMA7A for 4 hours were quantified by densitometry (n≥3). (TIF) [file pone.0146930.s006.TIF]

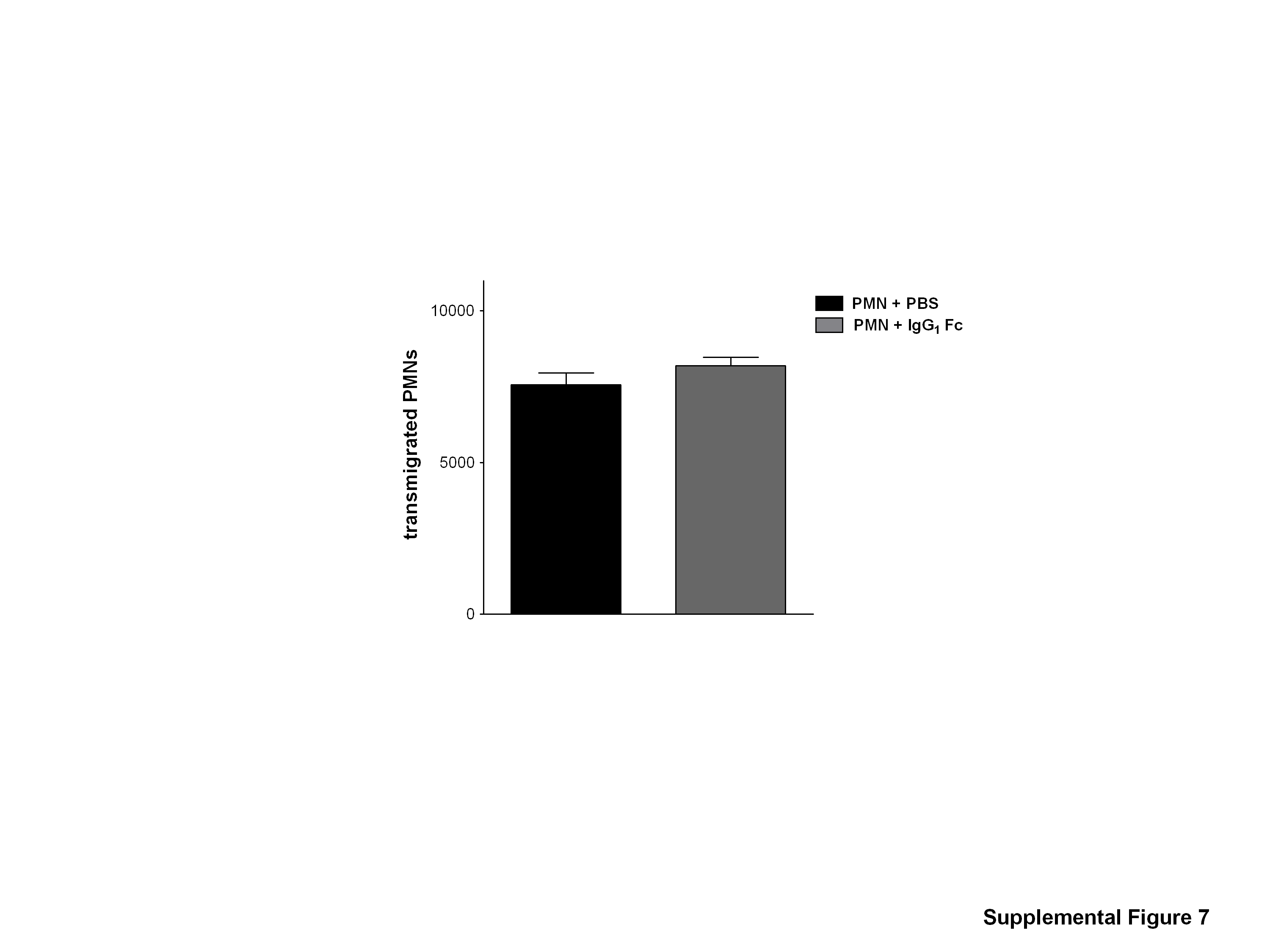

Supplement: S7 Fig — The migration of neutrophils was measured after 90 min (n = 14). (TIF) [file pone.0146930.s007.TIF]

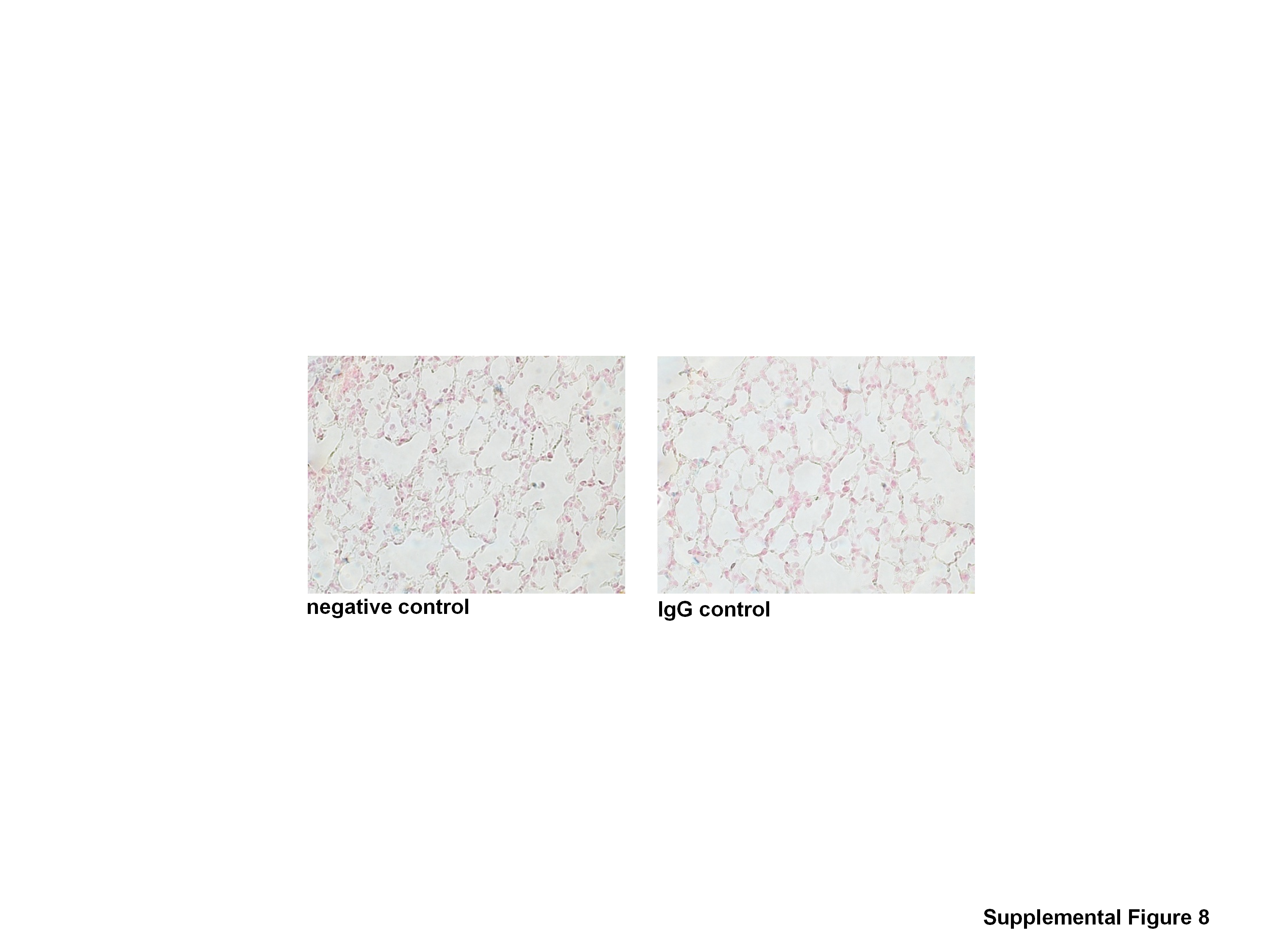

Supplement: S8 Fig — (TIF) [file pone.0146930.s008.TIF]
